# Multi-Temporal Hyperspectral Classification of Grassland Using Transformer Network

**DOI:** 10.3390/s23146642

**Published:** 2023-07-24

**Authors:** Xuanhe Zhao, Shengwei Zhang, Ruifeng Shi, Weihong Yan, Xin Pan

**Affiliations:** 1 College of Computer and Information Engineering, Inner Mongolia Agricultural University, Hohhot 010018, China; 2 College of Water Conservancy and Civil Engineering, Inner Mongolia Agricultural University, Hohhot 010018, China; 3 Center of Information and Network Technology, Inner Mongolia Agricultural University, Hohhot 010018, China; 4 Institute of Grassland Research of CAAS, Hohhot 010010, China

**Keywords:** multi-temporal, hyperspectral classification, grassland, transformer network

## Abstract

In recent years, grassland monitoring has shifted from traditional field surveys to remote-sensing-based methods, but the desired level of accuracy has not yet been obtained. Multi-temporal hyperspectral data contain valuable information about species and growth season differences, making it a promising tool for grassland classification. Transformer networks can directly extract long-sequence features, which is superior to other commonly used analysis methods. This study aims to explore the transformer network’s potential in the field of multi-temporal hyperspectral data by fine-tuning it and introducing it into high-powered grassland detection tasks. Subsequently, the multi-temporal hyperspectral classification of grassland samples using the transformer network (MHCgT) is proposed. To begin, a total of 16,800 multi-temporal hyperspectral data were collected from grassland samples at different growth stages over several years using a hyperspectral imager in the wavelength range of 400–1000 nm. Second, the MHCgT network was established, with a hierarchical architecture, which generates a multi-resolution representation that is beneficial for grass hyperspectral time series’ classification. The MHCgT employs a multi-head self-attention mechanism to extract features, avoiding information loss. Finally, an ablation study of MHCgT and comparative experiments with state-of-the-art methods were conducted. The results showed that the proposed framework achieved a high accuracy rate of 98.51% in identifying grassland multi-temporal hyperspectral which outperformed CNN, LSTM-RNN, SVM, RF, and DT by 6.42–26.23%. Moreover, the average classification accuracy of each species was above 95%, and the August mature period was easier to identify than the June growth stage. Overall, the proposed MHCgT framework shows great potential for precisely identifying multi-temporal hyperspectral species and has significant applications in sustainable grassland management and species diversity assessment.

## 1. Introduction

Grassland is an important natural barrier to maintaining the terrestrial ecological environment and is the basis of livestock production [1]. In recent years, grassland degradation has been a prominent problem confronting countries around the world [2,3]. The accurate and rapid assessment of species distribution provides powerful monitoring data for the scientific detection and analysis of grassland, which is helpful for realizing the intelligent management of grassland and to further prevent degradation. These processes must be performed in in situ assessments by manually collecting samples and studying the changes over time, which is inefficient in large areas of grassland considering manpower constraints. Multi-temporal analysis is a valuable technology that enables the monitoring of dynamic changes over time in various applications, such as crop monitoring [4,5], early drought warnings [6,7], land use and land cover assessments [8], crop classification [9], and the detection of plant species [10]. It is mainly driven by satellite data of Landsat and MODIS and their long-term operation [11]. Multi-temporal hyperspectral data are a promising tool for precision agriculture, hydrogeology, urban planning, and environmental monitoring and have been extensively utilized in numerous scholarly works (e.g., [12,13,14,15,16]). Consequently, determining multi-temporal hyperspectral data for grassland classification is imperative for estimating losses in ecology, predicting yields, and improving grassland management for herdsmen in animal husbandry production.

Hyperspectral imagery (HSI) has hundreds of continuous narrow spectral bands, which provide more detailed measurements than traditional multi-spectral imagery [17,18]. Each pixel in HSI is characterized by a wealth of spectral information, which opens a wide range of possibilities for distinguishing objects [19]. Many studies of grassland classification have been carried out worldwide using HSI technology [3,20,21]. Multi-temporal hyperspectral data refer to the time series of HSI, which allow for the monitoring of vegetation evolution over the course of a growing season or between years [10,22].

In terms of vegetation detection, Somers et al. performed a study with multi-temporal hyperspectral mixture analysis and feature selection for invasive species’ mapping in rainforests and evaluated the potential of a multi-temporal Multiple Endmember Spectral Mixture Analysis (MESMA), which reached 0.78 kappa [10]. McCann et al. studied the multi-temporal hyperspectral data of mixed agricultural and grassland regions for anomaly detection and performed a histogram classification of biophysical parameters, revealing the effectiveness of data over a period of time for quantitative comparison [11]. Kluczek et al. studied multi-temporal remote-sensing images for the mapping of mountain plant communities using random forest (RF) and support vector machine (SVM) classifiers that obtained a 76–90% F1-score [23]. In terms of grassland classification, Möckel et al. established partial least-squares discriminant analysis models to determine grazed vegetation belonging to different grassland successional stages, and the overall classification accuracy was 85% [24]. Marcinkowska-Ochtyra et al. used the RF algorithm to identify two grass species, *Molinia caerulea* and *Calamagrostis epigejos*, in different growth stages. For *Molinia caerulea*, the highest median F1 was 0.89, and for *Calamagrostis epigejos*, it was 0.73 [25]. At present, the study of grassland classification based on multi-temporal hyperspectral data is relatively rare.

The above research has made wide use of hyperspectral sensors with varying resolutions that offer different degrees of detail, but it has not produced suitable accuracies when attempting to identify grass species. The development of classification methods in this field has mainly involved machine learning using RF, and feature extraction relies on the domain knowledge and engineering experience of human experts [25,26,27,28]. Over time, convolutional neural networks (CNN) have shown prominence in HSI classification tasks [29,30], but they cannot sufficiently mine the sequence attributes of spectral signatures, hindering further performance advances [31]. Recently, a novel deep-learning mechanism of the transformer network [32] was proposed to solve classification tasks from a time series data perspective.

Transformer networks have received a high degree of attention due to their excellent performance in natural language processing (NLP) [33], computer vision (CV) [34], and other disciplines [35]. A transformer network was proven to be effective in hyperspectral classification tasks owing to its prominent capability in capturing long-term dependency [35]. Peng et al. studied a spatial-spectral dual-branch sequence network for HSI classification based on a transformer network and obtained the highest classification accuracy of 99.82% [36]. Zhang et al. studied a convolution transformer mixer for HSI classification, and the overall classification accuracy was improved by 0.31–0.75% [37]. Qing et al. studied the transformer model for HSI classification, relying on the self-attention mechanism; the method achieved an average accuracy of above 98.95% [38]. Yang et al. studied the hyperspectral image transformer (HiT) classification network and showed the superiority of the HiT network over the state-of-the-art CNN-based methods [31].

Transformer networks have shown powerful processing capabilities in hyperspectral classification, particularly for long-range sequence features. Therefore, in this work, we attempt to explore the prospect of this network in the field of multi-temporal hyperspectral data and introduce it to the task of grassland classification. In addition, the multi-temporal data of grasslands in this paper contain different grassland succession stages and range between years. With the use of a multi-temporal dataset, the complexity of the study increases.

Based on the above-mentioned analysis, the innovative element of this study is its proposal of the multi-temporal hyperspectral classification of grasslands using a transformer network (MHCgT). The main objective is to evaluate the potential of combining a time series of HSI and an automated feature selection technique in the network for grass species’ detection. Specifically, the multi-temporal analysis uses plant phenology, and the feature selection implements automatic recognition of the best time and the prime spectral feature set of corresponding species, which optimizes the separability among objects. A modified transformer-based approach with spectral attention blocks is tested on a time series of HSI covering a grassland area of Inner Mongolia in northern China. The ultimate goal of the paper is to aid the classification of species in this complex grassland ecology and explore the optimum identification period, proposing a model approach to be used in other grassland regions. 

The remainder of this paper is organized as follows. Section 2 introduces the study area, experimental data, and the proposed MHCgT method. Section 3 presents an experimental analysis of MHCgT. Section 4 discusses the performance of the proposed method versus five current methods, and Section 5 provides a summary.

## 2. Materials and Methods

### 2.1. Study Area

**Figure 1:**
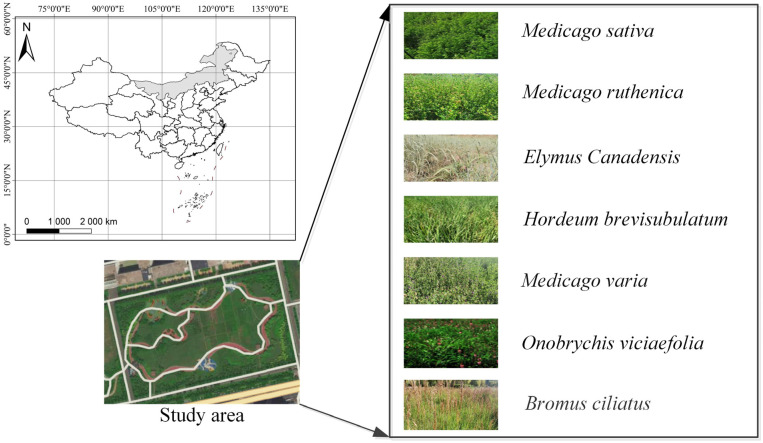
Location of the study area within China (Inner Mongolia Autonomous Region, China) and the grass species located in the study site over a topographic map (https://ditu.amap.com, accessed on 23 January 2023).

The study site was in Inner Mongolia Autonomous Region, China. It is a vast region located at a high latitude, and the landform is dominated by the Mongolian Plateau. The climate in this region is characterized as a temperate continental monsoon climate. The annual precipitation is 100–500 mm, mainly occurring from May to September. The size of the grassland in Inner Mongolia Autonomous Region is approximately 880,000 square kilometers, which ranks at the top in China and serves as an important natural ecological barrier in northern China [39]. The vegetation in the experimental area is mainly typical grassland plants (Figure 1).

### 2.2. Framework

The MHCgT network implementation details were implemented on the Keras framework, using a NVIDIA GeForce RTX 3090 GPU with 24 GB RAM. Our aim was to test the performance of a modified transformer-based deep-learning network for individual species’ identification in a northern grassland using multi-temporal hyperspectral data, with a special focus on Asian forage. Our task was divided into four separate subtasks:

(i) Collect field grassland species by multi-temporal hyperspectral data;
(ii) Extract spectral characteristics of multi-temporal grassland;
(iii) Utilize these data to construct the MHCgT network;
(iv) Optimize the network by performing an iterative accuracy assessment.

### 2.3. Data Acquisition

The grassland samples were scanned by a Hyperspectral Imager (HyperSpec©PTU-D48E, Golden Way Scientific, Beijing, China). The spectral wavelength range of the imager is 400–1000 nm, with a total of 125 bands. The exposure time of the Andor Luca detector was set at 10 ms, the platform moving length at 35°, and the spectral resolution at 4.8 nm. 

Hyperspectral imaging allows for the recognition of specific characteristics of individual species but requires appropriate data collection periods. A relevant study indicated that the best results are achieved in late summer and early autumn, because, during this period, plant species have typical characteristics in color and morphology [23]. Thus, in this experiment, multi-temporal hyperspectral images of grassland were collected at the end of June and the beginning of August 2020 and 2021, respectively. A total of 7 typical grass species and 84 sample areas were set up from different angles. The average reflectance spectrum of the hyperspectral images was extracted through the regions of interest (ROI). Of the 7 species, 600 spectral curves were collected for each class in every period, excluding some spectral data that were uneven and unrepresentatively distributed with the actual experiment, and finally, 16,800 valid pieces of spectral data were obtained (Table 1). Subsequently, the Savitzky–Golay (S-G) smoothing filter algorithm was used to preprocess grassland hyperspectral data to better extract spectral features and reduce noise impact.

### 2.4. Object-Based Classification

**Figure 2:**
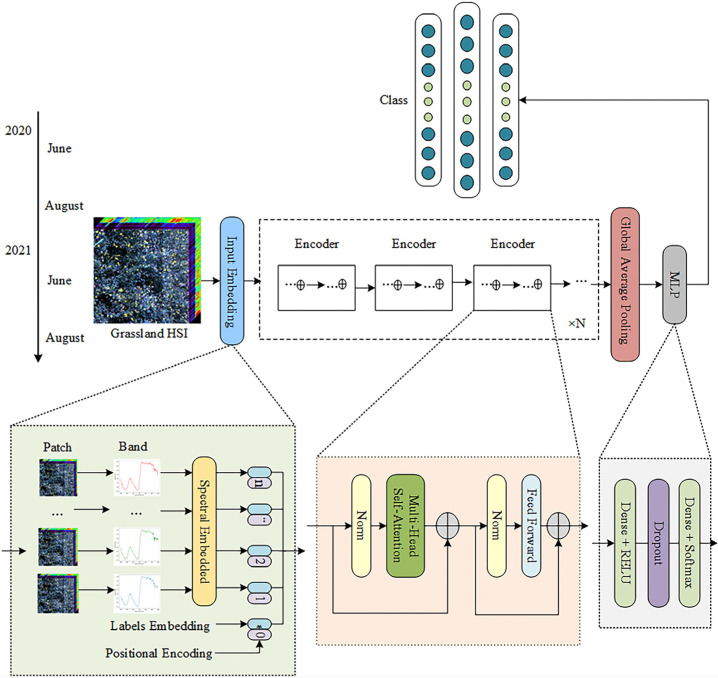
The overall architecture of the proposed MHCgT framework.

In this section, we propose a multi-temporal hyperspectral classification of grassland based on the transformer network (MHCgT), which realizes the application of the transformer structure in multi-temporal hyperspectral classification scenarios. On this basis, the function and importance of the network, the multi-head self-attention mechanism, the encoder block, and the classification layer used in MHCgT are analyzed and explained. The detailed architecture of the MHCgT framework is depicted in Figure 2, which includes three contributions in terms of model design and architecture:

1. Positional encoding is added to the grassland multi-temporal hyperspectral data to solve the problem of matching the position part of the transformer network with the time series scene.
2. The multi-head self-attention encoder block is employed to realize feature extraction and to process the remote dependence of spectral band information of hyperspectral data.
3. The hierarchical architecture of MHCgT generates a multi-resolution representation beneficial to the classification of the grass hyperspectral time series. And the encoder blocks are directly connected, effectively reducing the time and memory complexity.

#### 2.4.1. Positional Encoding

The model used for this project consisted of a transformer network. Transformer networks are based on a self-attention mechanism designed primarily to solve tasks in the field of NLP, as they perform well [32]. Recently, the application of a transformer model in the field of CV, called vision transformer (ViT) [34], has achieved excellent results in image classification, and to a certain extent exceeded the most advanced CNN model. Transformer networks have shown strong modeling ability for long-sequence data and are thus being used for multi-temporal hyperspectral classification.

Unlike NLP or ViT, transformer application in multi-temporal hyperspectral data has the important feature of a time series. Constructing an effective model of temporal dependency vis-à-vis seasonality or periodicity remains a challenge. Consequently, in the aspect of model design, positional encoding was added in the input embedding of the multi-temporal hyperspectral data to realize the adaptation of the position part of the normal transformer to the time series scene.

Specifically, the grassland multi-temporal hyperspectral dataset consists of multi-variable sequence information. The time series dataset is defined as shape tensor (N,S,M), where N is the number of samples in the dataset, S is the maximum number of time steps in all variables, and M is the number of variables processed in each time step. When M is 1, it is a single variable time series dataset. MHCgT utilizes the positional encoding added in the input embedding to model the sequence information [35]. The position embedding is a fixed value. For the feature map of multi-temporal hyperspectral grassland, it realizes the n-dimensional positional encoding method and changes the shape to meet the input of the model. This encoding contains the dimension vector of the specific position information in the spectrum and enhances the model’s input by injecting the spectrum’s sequence information.

#### 2.4.2. Multi-Head Self-Attention Mechanism

The transformer network uses an attention mechanism as the core construction model of the encoder-decoder and performs well [35]. The attention mechanism automatically and selectively focuses on specific information according to the situation, and it has been widely employed in NLP, image classification, and other fields [40,41]. Self-attention improves the attention mechanism to better capture data correlation. In this study, we employed the multi-head self-attention module, a variant of self-attention, to extract features. The multi-head self-attention mechanism is the key to the positive global modeling ability of MHCgT, which allows the model to process various information from different subspaces.

The multi-head self-attention groups the features in the channel dimension; each head is a group and conducts special attention for the group. Finally, the output is consolidated and calculated. Its expression is as follows:

(1) $$MultiHeadSelfAttn(Q,K,V)=Concat(head_{1},head_{2},\cdots ,head_{n})W{}^{\circ}$$

where n denotes the number of heads. Each head is concatenated to realize the calculation of multi-head self-attention. W° represents the linear transformation matrix. The head is based on the scaled dot-product attention that consists of query (*Q*), key (*K*), and value (*V*). First, calculate the dot-product of *K* and *Q* to form a dot-product matrix and normalize it. Obtain the attention weight score matrix through the Softmax layer. Then, multiply V to achieve self-attention. The specific calculation process is as follows:

(2) $$head_{i}=SelfAttn(QW_{i}^{Q},KW_{i}^{K},VW_{i}^{V})=A_{i}V$$

(3) $$A_{i}=Soft\max(\frac{Q_{i}K_{i}{}^{T}}{\sqrt{D_{k}}})$$

where $W_{i}^{Q},W_{i}^{K},W_{i}^{V}$ denote the mapping matrix of the *i*th head corresponding to the query, key, and value, respectively. $D_{k}$ represents the dimension of the vector *K*.

#### 2.4.3. Encoder Block

**Figure 3:**
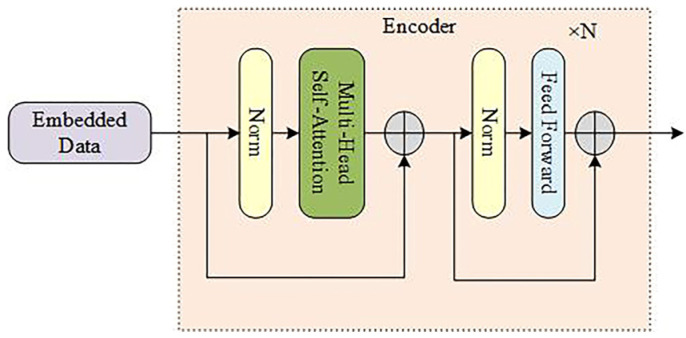
Structure of the encoder block.

The core process of our network involves the encoder block with multi-head self-attention, which successfully handles the long-distance dependence of the spectral band information of the multi-temporal hyperspectral image data. Further, in order to ameliorate the nonlinearity of the model, a feed-forward neural network is established, in which the spectral feature sequence output from the attention layer is passed The structure of the feed-forward part contains two convolution layers and embeds a RELU activation function. In this study, the encoder block mainly includes layer normalization (LN), the multi-head self-attention mechanism, and the feed-forward part, as shown in Figure 3. Significantly, MHCgT is composed of multiple encoder blocks, which together effectively mine the features with global dependencies of multi-temporal hyperspectral.

#### 2.4.4. Classification Layer

The model in this paper has an end-to-end network structure, with the multi-temporal spectral domain data as the input and the category label as the output. Grassland classification is completed by multilayer perceptron (MLP). MLP is the final layer structure for the MHCgT network, which is composed of two fully connected layers and a RELU activation function. Lastly, Softmax is used to obtain the class of the multi-temporal hyperspectral grassland. Additionally, the global average pooling operation is connected after the entire encoder block process, and the Dropout layer is introduced into the self-attention function, the feed-forward neural network, and MLP to prevent the depth model from over-fitting. The number of training sessions was set to 20 in each experiment. During the training process, the output model with the highest accuracy rate is used on the verification set. If the rate is consistent, the output model contains the smallest loss.

Owing to the transformer-based method requiring a large number of training samples [42], an ablation study of the percentage of training samples was carried out. We utilized the Stratified ShuffleSplit cross-validator to provide train/test indices and achieve data splits. The Stratified ShuffleSplit cross-validator object is a merge of StratifiedKFold and ShuffleSplit. It returns stratified randomized folds that retain the probability of samples in each category. The samples were randomly disordered, and the number of splitting iterations was set to 10. Additionally, we conducted a comparative analysis of MHCgT against five current methods, i.e., a convolutional neural network (CNN) [9], a recurrent neural network with long short-term memory (LSTM-RNN) [43], a random forest (RF) model [20], a support vector machine (SVM) model [23], and a decision tree (DT) model [21]. Each model undergoes the appropriate fine-tuning of parameters to achieve its optimal performance [9,20,21,23,43]. The consistency of parameter settings is maintained as much as possible in different models.

### 2.5. Accuracy Assessment

Classification accuracy and a confusion matrix are used to quantitatively evaluate the performance of the model. Accuracy means that the model correctly predicts the ratio of sample size to the total number of samples. Generally, the accuracy is proportional to the model effect. The index is calculated using true positive (TP), true negative (TN), false positive (FP), and false negative (FN) in the following way:

(4) $$\mathrm{Accuracy}=\frac{\mathrm{TP}+\mathrm{TN}}{\mathrm{TP}+\mathrm{TN}+\mathrm{FP}+\mathrm{FN}}$$

## 3. Results

### 3.1. Multi-Temporal Hyperspectral Data of Grassland

**Figure 4:**
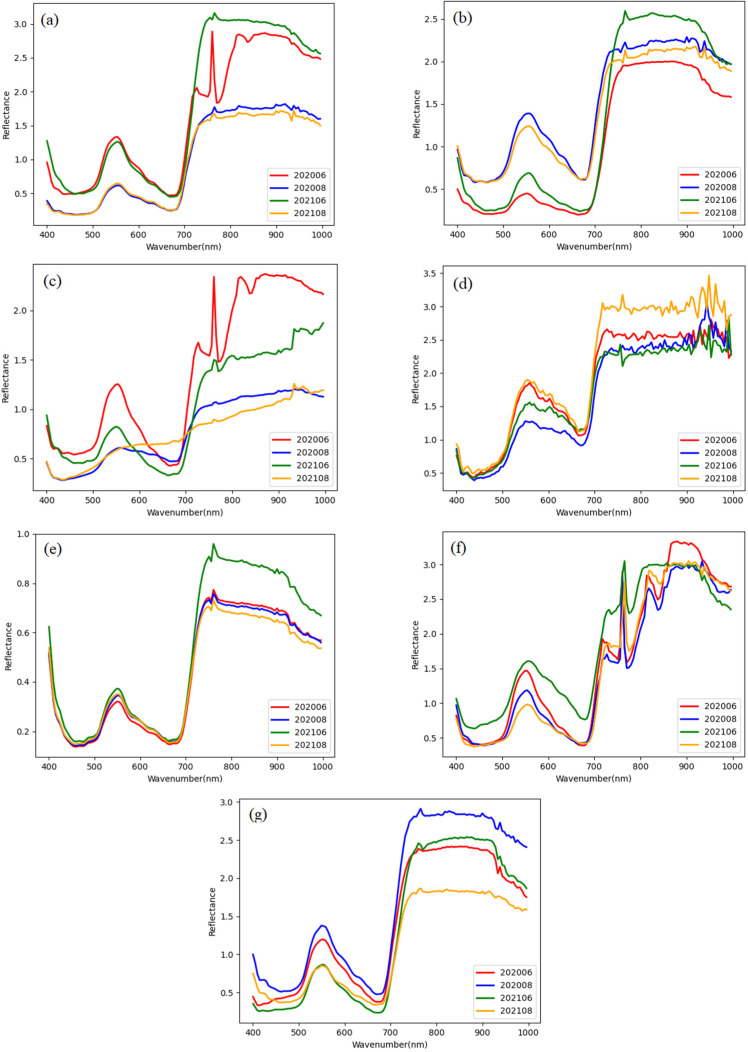
Multi-temporal hyperspectral data of seven grass species, namely, (**a**) Medicago sativa, (**b**) Medicago ruthenica, (**c**) Elymus canadensis, (**d**) Hordeum brevisubulatum, (**e**) Medicago varia, (**f**) Onobrychis viciaefolia, (**g**) Bromus ciliatus.

Hyperspectral imagery within the ROI on the sample was selected, which obtained the average reflectance spectrum with a band range of 400–1000 nm. Representative ROI were selected for each sample. The database contains seven species in June (growth period) and August (maturity period) of 2020 and 2021, respectively, 202006, 202008, 202106, and 202108, with a total of 16,800 spectral data. Figure 4 shows the multi-temporal hyperspectral data of the seven grass classes using 28 samples.

### 3.2. Classification Results

**Figure 5:**
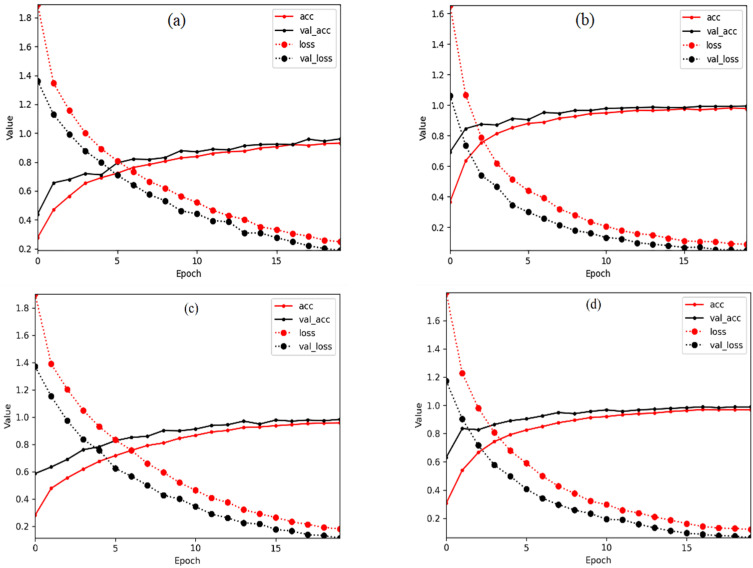
Relationship between epoch, accuracy, and loss in MHCgT network. (**a**) 202006, (**b**) 202008, (**c**) 202106, (**d**) 202108, acc: train accuracy, val_acc: validation accuracy, loss: train loss, val_loss: validation loss.

The interplay of each hyper-parameter of MHCgT was analyzed, and the optimal settings were obtained through multiple control experiments, with a total of 74,768 parameters. In Table 2, num heads indicates the number of attention heads, and ff dim stands for the hidden layer size in the feed-forward network inside the transformer network. We adopted the Adam optimizer with learning rate (lr) 1 × 10^−3^ and batch size 125. It should be noted that transformer-based methods can achieve excellent results when these parameters are set. We set the epochs on these four temporal datasets to 20 (Figure 5). Further, the EarlyStopping mechanism was added, and the patience is 10.

**Figure 6:**
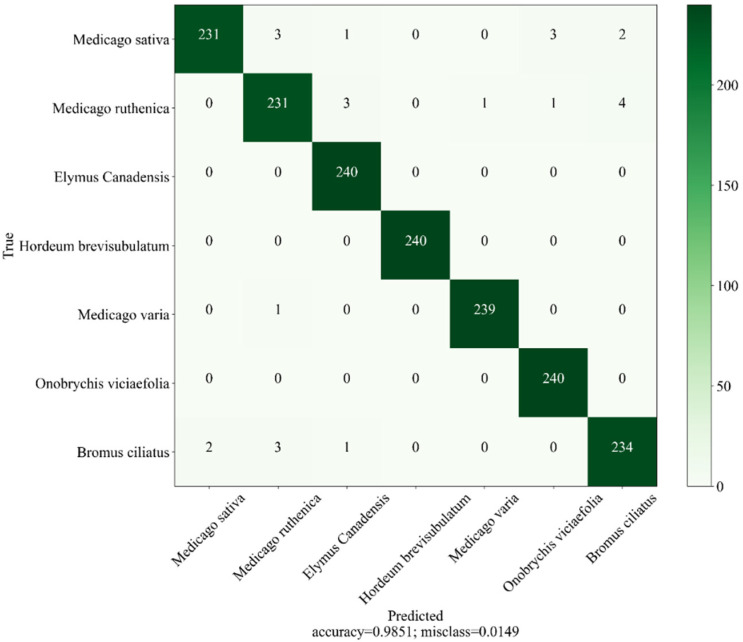
The confusion matrix is used for seven classifications. Rows represent actual classes, and columns represent prediction classes (test set 10%).

From the overall perspective of Figure 5, the accuracy of the test set slightly higher than the training set, whereas the loss is the opposite, indicating that the MHCgT network performs well in the training set and has a certain generalization ability. Figure 6 shows the confusion matrix as the best result of MHCgT for grassland multi-temporal hyperspectral classification. Table 3 is the identification results of single species during different periods. 

### 3.3. Ablation Studies

An ablation study was conducted on the percentage of training samples. We conducted extensive experiments on the four time-phase hyperspectral datasets, varying the training samples from 10% to 90% at intervals of 10%. The MHCgT was run five times. Table 4 reports the average results of the accuracy achieved by the proposed MHCgT.

Moreover, we conducted a comparative analysis of MHCgT against five current methods, i.e., CNN, LSTM-RNN, RF, SVM, and DT (Table 5). The ratio of the test set is 10%, the number of selected random items in each class is 1680, the epoch is 20, the batch size is 125, C is 1.0, and the max depth is 10. The result is the average of five experiments, with two decimal places for each one.

## 4. Discussion

### 4.1. Multi-Temporal Hyperspectral Analysis

Multi-temporal hyperspectral data contain hundreds of spectral bands and rich temporal information. In our case, the application of 125 original spectral bands and four pieces of time series information of two growth stages in two years was used to achieve efficient grassland classification and explore the optimum identification period. Firstly, the spectral signature of these grass classes follows a similar trend, with certain inter-class similarity. Secondly, each class covers different reflectivity based on these 16,800 samples. This means that these grass classes have a high standard deviation, resulting in a wide overlap between them, that is, all spectra are interwoven. Thirdly, the average reflectance spectral curve of each species has differences during multiple phases, where the peak/trough values of reflectivity are different under the same positions. Analyzing the influence of individual years and succession stages of data acquisition, it is difficult to see general rules in the case of classification results due to environmental conditions, e.g., weather, precipitation, and soil moisture, between different years. Each of the analyzed succession stages was characterized by the unique growth cycle of vegetation. The color and morphological elements of species are different in the growing season and the mature season, which further increases the intra-class differences and significantly affects the ability to distinguish individual communities. In Figure 4, the different average reflectance spectra of grassland samples during succession stages indicated that multi-temporal hyperspectral classification is feasible.

**Figure 7:**
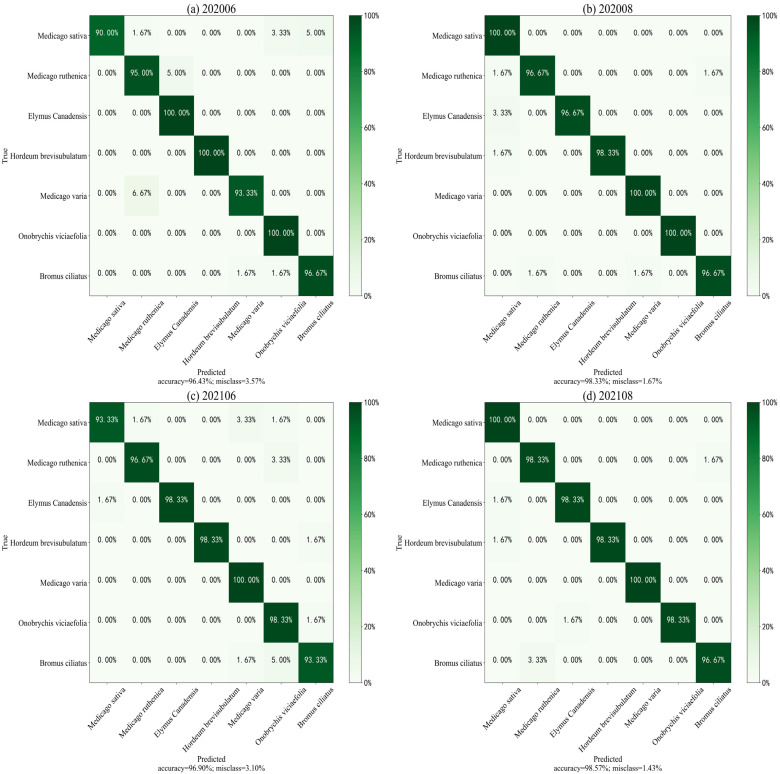
Confusion matrices of grassland multi-temporal hyperspectral data using MHCgT network (test set 10%). Rows indicate correct labels, and columns indicate predicted labels.

According to the growth stages of the analyzed species, comparing hyperspectral data from different time phases, an MHCgT deep-learning network is proposed to achieve single dataset and multiple dataset detection and to then point out the optimal time for corresponding species’ recognition (Table 3, Figure 7). Specifically, the classification accuracy of *Medicago sativa*, *Medicago ruthenica*, *Medicago varia*, and *Bromus ciliatus* is better in the August mature stage than in the June growth stage, with *Medicago sativa* and *Medicago varia* reaching a maximum of 1 in August. *Hordeum brevisubulatum* are easier to distinguish during June growth than August maturity. The accuracy of *Onobrychis viciaefolia* in June and August is the same, but in 2020, it is higher than in 2021, which may be due to differences in environmental factors, such as climate and precipitation, between different years. Most significant is that the average classification accuracy of the seven species reached over 95%, and the overall multi-temporal hyperspectral classification of grassland can achieve a satisfactory result of 98.51% (Figure 6).

### 4.2. Classification Method

It is noteworthy that the training samples affect the performance of the proposed MHCgT network (Table 4). The result shows that the classification accuracy gradually improves with varying numbers of training samples from 10% to 90%. When increasing the training samples from 10% to 50%, the accuracy is obviously improved. This demonstrates that the number of training samples also affects the performance of the proposed MHCgT network. When the training samples change from 60% to 90%, particularly in the range of 80–90%, which proves the stability of the MHCgT model. Overall, MHCgT has good adaptability in training and testing, and individual differences have a limited impact on the transfer ability of the model between subjects.

Regarding reference methods, MHCgT was compared with CNN, which performs well in hyperspectral classification; with LSTM-RNN, which is skilled in sequence data processing; and with SVM, RF, and DT, which are often applied in vegetation detection (Table 5). The transformer-based MHCgT utilizes a multi-head self-attention module to extract features. This mechanism overcomes issues with fixed sequence attributes related to the LSTM-RNN, realizes the parallel computation of multi-temporal data, and is able to capture long-sequence features surpassing the CNN. This module substantially promotes the development of multi-temporal hyperspectral data model and classification accuracy. MHCgT and LSTM-RNN, by their architecture, outperformed CNN, which is reflected in the research [43]. And owing to the powerful learning ability of the spectral sequential dimension, MHCgT produced better results than CNN by way of 97.92% versus 85.36%, respectively, in terms of accuracy on the multi-temporal hyperspectral dataset. This result was consistent with recently published studies [31]. Compared to SVM, RF, and DT, MHCgT is more exact, with an increase of 13.63% to 26.23%. Additionally, among previous studies on the identification of vegetation monitoring, attention can be paid to the type of plant communities, the number of classes, the applied algorithms, and the spectral range of the sensor (Table 6). The obtained average accuracy (97.92%) of MHCgT is quite comparable to that obtained by other authors. The accuracy of RF and SVM in the literature [23] is above 95%, which may be due to significant differences in characteristics between mountain forest and non-forest plant communities. Another noteworthy aspect is the number of categories identified. Increasing the number of species classes leads to confusion in spectral differences between categories and a reduction in accuracy [44,45]. Due to different sensors, the results of the same category and algorithm also differ [14,46]. Therefore, the type of sensor, species category richness, and algorithm selection all have a vital impact on the results of vegetation classification.

Analyzing the results obtained by MHCgT and five current algorithms on grassland multi-temporal hyperspectral data and comparing them with other authors, it should be considered that MHCgT achieved satisfactory performance (Table 5 and Table 6). The core components of this model are the positional encoding and the multi-head self-attention mechanism, which enhance the capabilities of model input matching and feature extraction, respectively. The model learns to automatically extract the key properties from the data in order to discern these among others. There are multiple encoder blocks that are ultimately exported in a fully connected network. The MHCgT has a hierarchical architecture, a direct connection between encoders, and no preprocessing steps, so it is an end-to-end lightweight deep network. This paper outlines two uses for multi-temporal radiometrically referenced hyperspectral data, i.e., multi-year classification and the detection of multiple growth periods, by constructing a MHCgT model, and it fully demonstrates the feasibility of the MHCgT model. Meanwhile, the use of a varying number of training sets to make MHCgT work efficiently further improves the adaptability of the network, enabling it to have better self-learning and self-tuning capabilities.

## 5. Conclusions

This study presents a novel approach (MHCgT) for grassland classification that applies a transformer network with multi-temporal hyperspectral images. Firstly, the hyperspectral imaging system used to collect multi-temporal grassland sample data. Next, an end-to-end MHCgT classification and recognition model is established for the collected multi-temporal hyperspectral data. Finally, multiple cross-comparison experiments are conducted to further verify the robustness and interpretability of the MHCgT model. The results showed that the MHCgT recognition effect, with 98.51% accuracy, is the best among five current methods, including CNN, LSTM-RNN, SVM, RF, and DT. In particular, the average classification accuracy of each species was above 95%, and the August mature period was easier to identify than the June growth stage. This indicates that the identification method used by combining hyperspectral imaging technology and a transformer depth network can accurately identify the components of multi-temporal grassland, including the growth and maturity phases of grass communities and multi-year information. The model provides a non-destructive and effective detection method for grassland management. Future work will expand upon the sample type and temporal data, attempting to identify more different species of grassland and to optimize the model to reduce computational complexity.

## Figures and Tables

**Table 1:** Information on multi-temporal hyperspectral grassland sample species.

Class	Name	2020		2021		Samples
		June	August	June	August	
1	*Medicago sativa*	600	600	600	600	2400
2	*Medicago ruthenica*	600	600	600	600	2400
3	*Elymus Canadensis*	600	600	600	600	2400
4	*Hordeum brevisubulatum*	600	600	600	600	2400
5	*Medicago varia*	600	600	600	600	2400
6	*Onobrychis viciaefolia*	600	600	600	600	2400
7	*Bromus ciliatus*	600	600	600	600	2400
Total	-	4200	4200	4200	4200	16,800

**Table 2:** Parameter information of the MHCgT network.

Parameter	Setting	Parameter	Setting
Num heads	8	Lr	1 × 10^−3^
Ff dim	64	Beta 1	0.9
Num transformer blocks	4	Beta 2	0.98
Mlp units	125	Epsilon	1 × 10^−9^
Mlp dropout	0.4	Batch size	125
Dropout	0.25	Epochs	20

**Table 3:** Classification accuracy (%) of grassland multi-temporal hyperspectral data using MHCgT network.

Class	Name	2020		2021		Average Accuracy
		June	August	June	August	
1	*Medicago sativa*	90	100	93.33	100	95.83
2	*Medicago ruthenica*	95	96.67	96.67	98.33	96.67
3	*Elymus Canadensis*	100	96.67	98.33	98.33	98.33
4	*Hordeum brevisubulatum*	100	98.33	98.33	98.33	98.75
5	*Medicago varia*	93.33	100	100	100	98.33
6	*Onobrychis viciaefolia*	100	100	98.33	98.33	99.17
7	*Bromus ciliatus*	96.67	96.67	93.33	96.67	95.84
-	-	96.43	98.33	96.90	98.57	-

**Table 4:** Classification results of different training proportions.

Training Sets	Training Samples	Testing Samples	Loss	Accuracy
10%	1680	15,120	1.3995	0.5086
20%	3360	13,440	1.0376	0.6305
30%	5040	11,760	0.7957	0.7413
40%	6720	10,080	0.5902	0.8282
50%	8400	8400	0.3303	0.9181
60%	10,080	6720	0.2479	0.9331
70%	11,760	5040	0.1538	0.9603
80%	13,440	3360	0.1164	0.9743
90%	15,120	1680	0.0829	0.9792

**Table 5:** Experimental evaluation of multi-temporal hyperspectral data of grassland classification against five current methods, highlighting the effectiveness of the proposed MHCgT network.

Method	MHCgT	CNN	LSTM-RNN	SVM	RF	DT
Accuracy (%)	97.92	85.36	91.50	84.29	82.56	71.69

**Table 6:** Comparison of the obtained results with those reported in the literature. Explanations: 3D-CNN—3D convolutional neural network, ANN—artificial neural network, RF—random forest, DT—decision tree, SVM—support vector machine.

Author	Spectral Range	No. of Classes	Object of Classification	Algorithm	Accuracy (%)
Our results	400–1000 nm	7	Grass species	MHCgT	97.92
Kupková et al. [44]	400–2500 nm	7	Mountain vegetation communities	SVM	84.3
Melville et al. [20]	600–875 nm	4	Grassland communities	RF	93
Yang et al. [21]	400–1000 nm	3	Desert steppe species	DT	87
Kluczek et al. [23]	416–995 nm	13	Mountain forest and non-forest plant communities	RF	98.5
	954–2510 nm			SVM	95.3
Mäyrä et al. [45]	406–995 nm	4	Tree species	3D-CNN	87
				ANN	81.7
	956–2525 nm			SVM	82.4
				RF	70.3
Zagajewski et al. [46]	413–2440 nm	4	Mountain forest	SVM	87
				RF	83
				ANN	84

## Data Availability

Not applicable.
